# Pesticide Residue Analysis of Khat Leaves and Health Risks among Khat Chewers in the Amhara Region, Northwestern Ethiopia

**DOI:** 10.1155/2021/4680573

**Published:** 2021-03-24

**Authors:** Seyfe Asrade Atnafie, Niguse Yigzaw Muluneh, Kefyalew Ayalew Getahun, Asegedech Tsegaw Woredekal, Wubayehu Kahaliw

**Affiliations:** ^1^Department of Pharmacology, School of Pharmacy, College of Medicine and Health Sciences, University of Gondar, Gondar, Ethiopia; ^2^Department of Psychiatry, School of Medicine, College of Medicine and Health Sciences, University of Gondar, Gondar, Ethiopia

## Abstract

**Introduction:**

Farmers use a large number of pesticides to increase the productivity of Khat cultivation. Since Khat is consumed by many Ethiopians as a habit, there may be a significant concentration of pesticide residues in Khat leaves which result in adverse effects among the exposed population. Excess use of pesticides in Khat causes cancer, genotoxicity, and endocrine disturbances. The current study aimed to determine the concentration of selected pesticide residues on Khat leaves samples and to estimate the possible health risk on consumers.

**Methods:**

Thirty Khat samples were collected from Khat cultivators in the Amhara region, Ethiopia. Pesticide residue extraction and cleanup of Khat samples were performed using the QuEChERS method. Data were also collected about personal variables and Khat consumption habits. The pesticide concentrations were determined and the health risks were estimated.

**Results:**

Profenofos, dimethoate, and chlorpyrifos were detected in 6 (20%), 24 (80%), and 28 (93.3%) Khat samples, respectively. However, diazinon was detected in two samples and endosulfan I was not detected. The average concentration of chlorpyrifos, dimethoate, profenofos, and diazinon in Khat samples was 75.39 ± 20.26, 130 ± 48.94, 242.45 ± 119.79, and 81.5 ± 21.0 *μ*g/kg, respectively. The HQ for diazinon was greater than 1 which shows the chronic health risks associated with intake of diazinon among Khat chewers. The acute health risks associated with intake of profenofos and diazinon were minimal. *Discussion*. Pesticide residue exposure is a risk factor for cardiac complications, reduction in antioxidant defense system, and developing neurological symptoms. All the pesticides were higher than the European Union Maximum Residue Levels (EU-MRL). Diazinon has the potential to cause higher chronic health risks. Chronic Khat chewers in the region may face potential health risks from pesticide residues. Awareness creation among people in the study area is necessary to avoid the adverse health effects of pesticide residues.

## 1. Introduction

Khat (*Catha edulis*) is an evergreen, mild-narcotic, flowering tree that mostly grows in East Africa. It was first cultivated in Ethiopia and Yemen. Khat leaves are chewed as a stimulant in many countries, by 5 to 10 million people every day mainly in the Middle East and Eastern Africa [1–3]. The plant is a controlled substance in some western countries, while it is legal in Djibouti, Kenya, Ethiopia, Somalia, and Yemen. Khat is a highly valued export commodity in Ethiopia [4].

In Ethiopia, the overall prevalence of Khat chewing was 15.3% from the Ethiopian demographic and health survey (EDHS). In the Amhara region, the reported prevalence of Khat chewing from the demographic health survey was 7.8% of the total population [5].

Farmers in several areas of Ethiopia reported that they often use DDT (dichlorodiphenyltrichloroethane) and other pesticides either alone or mixed with other toxic pesticides to kill pests of Khat. A study conducted on Khat expansion in Wondogenet, Southcentral Ethiopia, found that Khat growing farmers use different types of toxic pesticides as an insect controller and they spray it about 1-2 times per week on Khat [1]. Similarly, studies conducted on the status of pesticides in other parts of Ethiopia also evidenced the use of toxic pesticides for Khat growing purposes [6, 7].

Using pesticides as Khat growing chemicals can cause deleterious effects on human health and the environment [8]. High doses and chronic use of Khat can cause more serious adverse neurological, psychiatric, cardiovascular, dental, gastrointestinal, and genitourinary effects [9]. Furthermore, the extensive use of pesticides boosts the adverse effects of Khat chewing and environmental contamination. Excess use of pesticides in Khat causes cancer, genotoxicity, and endocrine disturbances. Pesticide-treated Khat consumption was also reported to have association with the development of non-insulin-dependent diabetes mellitus [10, 11].

Higher consumption of Khat in Ethiopia has led to increasing demand, and this, in turn, needs extensive use of pesticides to increase production; 95% of Khat farmers in Yemen haphazardly use hazardous and unofficially controlled pesticides for the Khat trees, risking the health of Khat chewers. The toxicity of various pesticides, including organochlorines and organophosphates (OPs), has been reported [12].

World Health Organization (WHO) classified pesticides like DDT, chlorpyrifos, diazinon, dimethoate, and profenofos under class II (moderately hazardous). The LD50s of these pesticides in rats are between 50 and 2000 mg/kg from oral exposure and between 200 and 2000 mg/kg from dermal exposure [13].

There is a lack of a population-based study about the dangers of using Khat and its psychoactive constituents and additional toxic pesticide residues in cultivating countries, such as Ethiopia [14]. There is a need for routine and systematic research of Khat-using populations in respect of morbidity arising from the use of pesticides in the cultivation of Khat [15]. Despite the fact that continuing application of pesticides for Khat cultivation by local farmers' results in the deposition of high concentrations of pesticide residues on Khat plants [16], the concentration of the pesticide residues on the leaves is not well documented. Moreover, the level of human exposure and health risk of these pesticides from consumption of the plant has never been quantified and reported in Amhara region, Ethiopia. The health risks of pesticides among chronic Khat chewers in Ethiopia are assumed to be magnificent. The present study is, therefore, the first attempt in the region intended to determine the pesticide exposure level and estimate the possible health risks of pesticide residues on Khat.

## 2. Materials and Methods

### 2.1. Study Design and Period

A cross-sectional study was employed in which Khat consumption practice was assessed. Pesticide residue concentration on Khat was also determined. Human exposure status and possible health risks of consumers from pesticide exposure were estimated in the Amhara region. The data was collected from February 24 to April 15, 2019.

### 2.2. Study Areas

**Figure 1:**
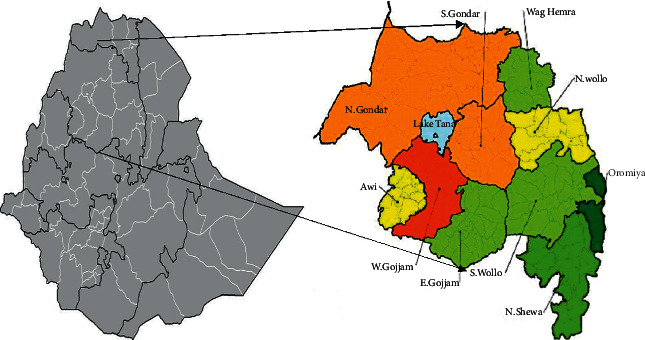
Khat consumption survey study areas in the Amhara region, Northwest Ethiopia, 2019 [18, 19].

The Khat samples for pesticide residue analysis were collected from local markets for the distribution of Khat throughout the Amhara region, Northwest Ethiopia (Figure 1). The local markets are found in rural kebeles around Enfranz, Kulkualber, Garno, Gulagulma, Haik, Kemissie, Gonbat, Sebatamit, Negdi, and Yiloma. These Khat cultivation areas are chosen as these are the main Khat cultivating and distribution sites in the Amhara region. The altitude of the study area makes it suitable for the cultivation of cash crops such as Khat [15].

Khat consumption survey was conducted among Khat chewers in urban and semi-urban areas in North Gondar, West Gojjam, and South Gondar zones of the Amhara region. These zones are purposely selected as the zones are found in the main Khat cultivation and transport corridor of the Amhara region. Transportation is the main barrier to the increased prevalence of Khat chewing as Khat is a perishable commodity. Gondar, Enfranz, Debretabor, Addis Zemen, Bahirdar, Merawi, and Tisabay towns were selected from the three zones. According to the 2015 report of the Central Statistical Agency (CSA), the projected total population of the Gondar, Enfranz, Debretabor, Addis Zemen, Bahirdar, Merawi, and Tisabay towns was 323900, 9162, 87100, 25300, 243300, 29300, and 27200, respectively [17]. 

### 2.3. Sample Size

The number of Khat chewers included in the current study was calculated using single population proportion formula with the following assumptions: confidence level of 95%, *z* = 1.96, significance level *α* = 5%, degree of precision, *w* = 0.04, and expected prevalence of *P* = 50% (since the Khat consumption practice and concurrent exposure to pesticides in the Amhara region are unknown). The calculated sample size after adding a 5% non-response rate was 508.

### 2.4. Sampling Techniques and Procedures

Proportionally allocated and randomly selected Khat chewers were interviewed for Khat consumption practice in 7 towns of the Amhara region. Face-to-face interviews were done by trained data collectors using a structured and pretested questionnaire.

### 2.5. Study Variables

Dependent variables for the assessment of health risks include estimated daily intake (EDI), estimated short-term intake (ESTI), and hazard quotient (HQ).

Independent variables include personal characteristics, Khat chewing practice measuring parameters, and the concentration of pesticide residues on Khat samples.

### 2.6. Inclusion/Exclusion Criteria

#### 2.6.1. Inclusion Criteria

Inclusion criteria were as follows: Khat chewers greater than 18 years, volunteer to participate in the study, and the representative Khat samples from volunteer Khat farmers.

#### 2.6.2. Exclusion Criteria

Exclusion criteria were as follows: Khat chewers less than 18 years of age and those who do not volunteer to participate.

### 2.7. Data and Khat Sample Collection and Analysis

Questionnaires for collecting data about Khat chewing practice were developed. The prepared questionnaires were pretested in Woreta town since the people in Woreta town have a similar socioeconomic status to other study areas. Necessary modifications from the pretest were made before proceeding to the actual data collection process. Health professionals were recruited for the data and Khat sample collection after taking the training. The collected data were checked for consistency, and completeness, organized, and entered into SPSS version 20.0. Descriptive statistics like percentages means and medians were calculated. Data are shown as mean ± standard error of the mean.

### 2.8. Reagents and Chemicals

Certified individual pesticides standards like diazinon (99.0%), chlorpyrifos (99.0%), dimethoate (98.5%), profenofos (98.5%), and endosulfan I (98.5%) were obtained from Jije Laboglass Pvt Ltd, Ethiopia. Individual stock solutions were prepared by dissolving an accurate weight of each pesticide in 100 ml of acetonitrile.

### 2.9. Preparation of Khat Samples

For the laboratory analysis, triplicate Khat samples from 10 different Khat cultivation areas were randomly collected with a total of 30 samples. The Khat samples were collected from volunteer Khat cultivators.

Half kg of Khat samples which contains the smoother and chewable part of Khat leaf was packed in labeled plastic bags and transported to the laboratory for analysis. Pesticide extraction and cleanup of Khat samples were performed using the QuEChERS method. The collected Khat samples were dried under shade and powdered. The laboratory analysis was done in Jije Laboglass Pvt Ltd, Ethiopia.

### 2.10. Sample Extraction/Partitioning

A 5.0 g amount of homogenized Khat sample was placed into a 50 mL centrifuge tube. A 10 ml aliquot of water was added to each tube using a dispenser and left to hydrate for thirty minutes after agitation for thirty seconds. A 10 mL aliquot of Acetonitrile was then added to each tube, capped, and agitated vigorously for one minute. A Bond Elut QuEChERS AOAC extraction salt packet, containing 6 g anhydrous MgSO4 and 1.5 g sodium acetate, was added directly to each tube. Tubes were sealed tightly and agitated vigorously for one minute to ensure that the solvent interacted well with the entire sample and crystalline agglomerates were broken up sufficiently. Finally, sample tubes were centrifuged at 4,500 rpm for 5 minutes.

### 2.11. Dispersive SPE Cleanup

A 2 mL aliquot of the upper acetonitrile layer was transferred to a Bond Elut QuEChERS AOAC dispersive SPE 15 mL tube. The tube contained 400 mg PSA, 1200 mg of anhydrous MgSO4, 400 mg C18, and 400 mg GCB. Two ml of n-Hexane was also added to each tube. The tubes were capped tightly and vortexed for 1 minute. The tubes were then centrifuged with a standard centrifuge at 4,500 rpm for 5 minutes. Finally, an aliquot of the upper hexane layer was transferred to a 2 ml autosampler vial for injection on GC-MS [20].

### 2.12. GC-MS Type and Conditions

An Agilent technology 7890B gas chromatograph (GC) hyphenated with 5977B Agilent technology mass spectrometer (MS) detector with an electron impact (EI) ion source at 230°C was used to analyze pesticide residues. A GC column used in the present study was A DB-5MS (USA) ultra-inert capillary column (DB-5MS, 30 m × 0.25 mm × 0.25 *μ*m). The mobile phase (carrier gas) used in the analysis was helium gas (99.9% pure) with a flow rate of 1 mL/min. MassHunter software (Agilent Technology) was used to interpret instrumental responses.

The injection mode utilized during the whole analysis was splitless mode keeping the inlet temperature at 250°C; sample injection volume was 2 *μ*l. The oven temperature was programmed as follows: oven temperature: 100°C for 2 minutes, 100°C to 170°C at 15°C/min, 170°C to 220°C at 2°C/min, then 220°C to 279°C at 10°C/min.

Retention time and mass spectra were used to identify peaks after the acquisition of the total ion chromatogram. The mass-to-charge (m/z) ratio of ions was scanned in the range of m/z 50–550 to confirm the retention times of all analytes. All analytes were determined in selective ion monitoring (SIM) mode. The identity of pesticides was confirmed by selecting the most abundant target ions of each pesticide and two characteristic fragment qualifier ions. The selected and monitored characteristic ions for SIM mode detection were as follows: endosulfan I (quantifier: 195; qualifiers: 339 and 341), endosulfan II (quantifier: 337; qualifiers: 339 and 341), endosulfan sulfate (quantifier: 272; qualifiers: 387 and 422), diazinon (quantifier: 304; qualifiers: 179 and 137), chlorpyrifos (quantifier: 314; qualifiers: 196.9 and 257.9), dimethoate (quantifier: 87; qualifiers: 93 and 125), and profenofos (quantifier: 339; qualifiers: 337 and 374). Average peak areas of triplicate analysis were used for comparison.

## 3. Method Validation

Pesticides like diazinon, dimethoate, chlorpyrifos, endosulfan, and profenofos are currently used for Khat cultivation by farmers in the study area. Quantitative estimation of pesticide residues detected in Khat samples was done using a calibration curve prepared from the standard mixture of pesticides 10, 50, 100, 200, and 300 *μ*g/kg.

The method was validated by determining the limit of detections (LODs) and limit of quantifications (LOQs). LOD and LOQ were calculated as 3.3 and 10 times the standard deviation divided by the slope of the calibration curve of five blank pesticide samples on method part. Repeatability or intraday precision (at intervals of 4 hours within a day) of the method was evaluated by spiking blank Khat samples with 50 *μ*g/kg and 100 *μ*g/kg of standard pesticides. Determination of the recoveries of the analytes in both samples was done by comparing the concentration obtained [21].

Method validation indicated that the calibration curves for each analyte were linear in the concentration range of 10–200 ng/g. The determination regression coefficient (*R*^2^) values were between 0.996 and 0.999 for all pesticides. The LOD and LOQ were 0.076 and 0.23 mg/kg for chlorpyrifos, 0.193 and 0.584 mg/kg for dimethoate, 0.227 and 0.686 mg/kg for profenofos, 0.039 and 0.118 mg/kg for diazinon, and 0.145 and 0.439 mg/kg for endosulfan I. The percentage recoveries obtained were 101.2% for diazinon, 101.2% for chlorpyrifos, 102.9% for dimethoate, 105.2% for profenofos, and 99.4% for endosulfan. All pesticide residues have satisfactory precision with a relative standard deviation (RSD) of <20% (Table 1).

### 3.1. Health Risk Assessment

Estimates of exposure from Khat were derived from two distinct pieces of information: the amount of pesticide residue in Khat and the amount of Khat that a person consumes. The data from the daily amount of Khat chewed was used for estimating OP intake. Both chronic and acute risk assessments were calculated from the residues of pesticides in Khat.

### 3.2. Chronic Health Risk Assessment

The EDI was calculated and compared with the acceptable daily intake (ADI) for each pesticide. According to food and agricultural organization, ADI is the daily maximum intake of a pesticide over a lifetime that will not result in adverse effects at any stage in the human life span [22]. The EDI is calculated by multiplying residual pesticide concentration in Khat samples (*μ*g/kg) with the daily intake of Khat (kg/day) divided by the average body weight of Khat chewers (kg). The EDI will be expressed as *μ*g/kg/day [23].

### 3.3. Acute Health Risk Assessment

The acute reference dose (ARfD) is an estimate for an oral exposure of a pesticide residue for a short-term duration (24 hr or less). There is non-uniform exposure of pesticide residue from Khat exposure that may result in acute health risks in Khat chewers [24].

Equations:

(1) $$\begin{array}{c} \text{HQ}=\frac{\text{EDI}}{\text{ADI}},\\ \text{where}\\ \text{EDI}=\frac{\text{Average Concentration}\times \text{ingestion rate}}{\text{body weight}}, \end{array}$$

EDI: estimated daily intake, HQ: hazard quotient, and ADI: acute daily intake.

If the HQ is greater than 1, the residues in Khat have the potential to cause chronic health risk.

(2) $$\begin{array}{c} \text{aHI}=\frac{\text{ESTI}}{\text{aRfD}}\times 100\%,\\ \text{where}\\ \text{ESTI}=\frac{\text{highest residue level}\times \text{consumption}}{\text{BW}}, \end{array}$$

aHI: acute health risk index, ESTI: estimated short-term intake, and aRfD: acute reference dose.

If the aHI is greater than 100, the pesticide residues have the potential to cause acute/short-term health risks.

### 3.4. Data Quality Assurance

The data quality was maintained by collecting representative Khat triplicate samples and using validated analytical procedures. We also maintained and applied standard procedures and using analytical graded products.

### 3.5. Data Analysis

Data were entered and analyzed using SPSS version 20 statistical software. Descriptive statistics like percentages, means, and medians were calculated. Data are shown as mean ± standard error of the mean. Exposure analysis of the Khat chewers was conducted using the probabilistic exposure analysis method using Microsoft Excel 2010.

### 3.6. Ethical Consideration

Ethical clearance was obtained from the institutional review board of the University of Gondar with a reference number of O/V/P/RCS/05/484/2017 to conduct the study. Official letters were sent to the respective Zonal and Woreda health and agricultural bureau. Participants were briefed about the purpose of the study. Confidentiality was granted for information collected by keeping the privacy of the respondents while filling the questionnaire. Any personally identifiable information (such as names, addresses) was not entered into the database.

## 4. Results

### 4.1. Sociodemographic and Khat Chewing Characteristics

A total of 508 Khat chewers were included in the study with a response from 478 respondents (94.1%). From Khat chewing survey, the majority of the respondents (94.1%) were males. Around half of the respondents (46.7%) were young between 25 and 30. The average body weight of Khat chewers in the Amhara region was found to be 62.3 ± 0.41 kg. Less than half of the respondents (38.1%) chewed Khat for 6–10 years. Khat chewers chew Khat for an average period of 8.56 ± 0.26 years. The maximum number of years spent was 31 years and a minimum of 1 year. The average time that Khat chewers spent in a day was 179.7 ± 3.00 minutes. Most of them (60.9%) spent more than 180 minutes per day on Khat chewing. The majority of Khat chewers chew between 100 and 200 grams of Khat (56.5%) and chew Khat daily (57.5%). The maximum amount of Khat reported to be consumed by an individual was 500 g (Table 2).

### 4.2. The Occurrence of Pesticides in Khat

Among pesticide residues, profenofos, dimethoate, and chlorpyrifos were detected in 6 (20%), 24 (80%), and 28 (93.3%) Khat samples, respectively. However, diazinon was detected in two samples, and endosulfan I was not detected. The total pesticide residue concentration in triplicate Khat samples collected in different Khat cultivation areas of the Amhara region was variable. The average concentration of dimethoate detected in Khat samples was 130.0 ± 48.9 *μ*g/Kg. The maximum and minimum dimethoate residue concentrations were 463.5 *μ*g/kg and 34.2 *μ*g/kg, respectively. Chlorpyrifos was detected in all Khat samples collected in different areas of the Amhara region. The average concentration of chlorpyrifos in Khat samples was 75.4 ± 20.26 *μ*g/kg. The maximum and minimum concentrations of chlorpyrifos residue detected in Khat samples were 173.2 *μ*g/kg and 6.7 *μ*g/kg, respectively. The other pesticide residue detected was profenofos with an average concentration of 242.5 ± 119.8 *μ*g/Kg. The maximum and minimum profenofos residue concentrations were 449.7 *μ*g/kg and 34.7 *μ*g/Kg, respectively. The average diazinon residue concentration in Khat samples was 81.5 *μ*g/Kg. The maximum and minimum diazinon residue concentrations were 102.5 *μ*g/Kg and 60.5 *μ*g/Kg, respectively (Table 3).

In comparison with European Union Maximum Residue Level (EU-MRL), the average concentration of chlorpyrifos, dimethoate, profenofos, and diazinon residue levels was greater than the EU recommended standards for tea and herbal products [25].

### 4.3. Possible Health Risks of Pesticides

The EDI was calculated using average pesticide concentration and average chewed amount of Khat (200 g/day) divided by the average body weight of Khat chewers in the Amhara region (62.3 kg).

The HQ for diazinon was 1.31. The HQ for diazinon was greater than 1 which shows the chronic health risks associated with intake of diazinon among Khat chewers. The acute health risks associated with intake of profenofos and diazinon were minimal. Though aHI for chlorpyrifos and dimethoate was less than 100%, there is a possibility of acute health risks associated with intake of chlorpyrifos and dimethoate (Table 4).

## 5. Discussion

Pesticides are widely used to protect plants and crops in agriculture from pests and plant diseases. There are different categories of pesticides such as insecticides, fungicides, and herbicides. The European Union reported in 2010 that 208,000 tons of pesticides were used which are biologically active with specific inherent toxicity [15]. Recently, the use of pesticides has been increased. Therefore, there are many legal limits for pesticide residues in the plants set by the European Commission (EC) and United States, Department of Agriculture, to control pesticide usage who has reported that three million pesticide poisonings occur annually and result in 220,000 deaths worldwide. In public health safety and trade, pesticide residue determination in all food products, especially in raw vegetables and fruits, is a very demanding task [27]. In our study, the chronic health risks of dimethoate and diazinon were higher than the acceptable range. All the four pesticides detected in Khat samples were greater than the EU-MRL. In comparison with EU-MRL, the average concentration found in khat leaves was 7.54, 2.6, 4.85, and 1.63 times for chlorpyrifos, dimethoate, profenofos, and diazinon, respectively [24].

There were reports that Khat farmers do not follow the safety instructions during pesticide spraying [28]. A study from Haromaya Woreda, Ethiopia, indicated that some Khat farmers harvested a recently sprayed Khat for sale and self-consumption, potentially putting themselves and their consumers at higher risk of oral exposure to and ingestion of pesticides. Some Khat farmers prefer to use higher doses of pesticides than that recommended on the label [29].

Studies indicated that pesticide exposure is a risk factor for cardiac complications among Khat users. The presence of pesticides in Khat was also implicated in the inhibition of serum acetylcholinesterase activity and may contribute to the presence of high levels of free radicals and production of oxidants, responsible for a reduction in the antioxidant defense system [20]. Khat chewing had also a significant association with developing neurological symptoms [30].

Another study conducted in 2009 investigated the presence and level of pesticide residues in Khat samples collected from various parts of Ethiopia. The Khat samples had pp'-DDT concentrations ranging from 141.2 to 973.0 *μ*g/kg. Maximum concentrations were found between 240 and 1200 times the European Union maximum recommended levels for DDT in food (vegetables 10 *μ*g/kg, and cereals 50 *μ*g/kg) [28]. In a recent study on DDT, the concentration was 11.3 *μ*g/kg for p'p-DDT and 26 *μ*g/kg for the metabolite, p'p-DDE. The lower concentration of p'p-DDE showed the decreased application of p'p-DDE in Khat cultivation. Moreover, the higher concentration of the metabolite (p'p-DDE) compared to the parent compound (p'p-DDT) revealed the historical use of DDT in Southwestern Ethiopia [22]. Farmers in Amhara region, Northwestern Ethiopia, also reported historical use of DDT but no more applied for Khat cultivation. The main processor and marketer of DDT for malaria control in Ethiopia, Adami Tulu Pesticide PLC, has discontinued the production of DDT as of 2011 [31]. This is the main reason why DDT is not included in the analysis of pesticide residues in our study. Other pesticides like diazinon, dimethoate, chlorpyrifos, endosulfan, and profenofos are registered and widely used in Khat cultivation in Ethiopia.

Pesticide use in Ethiopia is at a tremendous increase for the past decade. At present, most agricultural pesticides used in Ethiopia are imported. In 2012, Ethiopia imported 3611.7 metric tons of pesticides [32]. Adami Tulu Pesticide PLC also formulated 2245 metric tons of pesticides in 2012 [31]. One of the reasons for the increased pesticide utilization may be Khat cultivation. Khat is an evergreen perennial shrub and pesticides are applied once or twice per week. Khat is a cash crop, which can bring substantial returns. The fresh, smooth, and marketable leaves of the Khat tree will not grow in sufficient amount if pesticides are not applied frequently. In general, Khat cultivation and market is directly dependent on the frequent application of pesticides [1, 33].

Dimethoate is an organophosphorus insecticide with contact and systemic action. It is used against a broad range of insects in agriculture and also for the control of the housefly [34]. Dimethoate was not in a detectable range in a study conducted in Yemen on Khat samples which are different from our finding in the current study. However, residual dimethoate has been found on Khat leaves produced in Yemen which is in agreement with our study [35]. In the current study, the average daily intake of Khat was 129.97 *μ*g/kg of body weight which is beyond the daily food intake (0.001 *μ*g/kg) [30]. Chronic dimethoate poisoning can lead to weakness, fatigue, slurred speech, and lack of coordination which are typical symptoms of cholinesterase inhibition. WHO has classified dimethoate as moderately hazardous. The half-life of dimethoate in different plants (including Khat) is between 2 and 5 days. Hence, Khat chewers might be exposed to residual dimethoate if they consumed Khat leaves within 2 to 5 days of spraying the pesticide [36].

Chlorpyrifos was detected in all Khat samples and this may be due to large distribution and persistence in Khat samples [37]. Chlorpyrifos is highly toxic to aquatic organisms and a potent developmental neurotoxin at low levels of exposure, below those that trigger fetal cholinesterase inhibition. Chlorpyrifos is an endocrine disrupter with anti-androgenic and estrogenic properties and reduces serum levels of cortisol and thyroid hormone [28]. Exposures in utero and in early childhood can lead to behavioral anomalies in adolescence and adulthood. Epidemiological studies in humans found delayed cognitive and psychomotor development and reduced IQ. Chlorpyrifos has been detected in human breast milk, cervical fluid, sperm fluid, cord blood, and the meconium of newborn infants [38].

The EPA's November 2016 assessment of chlorpyrifos found that multiple studies link low-level exposures during prenatal development and early childhood to disrupted brain development—leading to long-term mental and motor delays, lower IQ, poorer memory, and learning disabilities, as well as behavioral problems like attention deficit hyperactivity disorder (ADHD). The EPA further found that using chlorpyrifos on food crops results in unsafe exposures through contaminated food and drinking water [39].

## 6. Conclusion

The chlorpyrifos, dimethoate, profenofos, and diazinon pesticide residue concentration in Khat leaves was higher than the maximum residue level. Dimethoate and diazinon residue concentrations have a higher potential to cause chronic health risks. None of the pesticide residue concentrations are associated with acute health risks. Khat chewers in the region may face deleterious effects of pesticide residues along with adverse effects of Khat leaves. Awareness creation among people in the study area is necessary to avoid the adverse health effects of pesticide residues.

## Figures and Tables

**Table 1:** Method validation results in Khat residue analysis: recovery %, regression coefficient, limit of detection (LOD), and limit of quantification (LOQ).

Pesticides	*R* ^2^	Recovery (%)	LOD	LOQ	RSD
Chlorpyrifos	0.9996	101.2	0.076	0.231	6.78
Dimethoate	0.9977	102.9	0.193	0.585	12.85
Profenofos	0.9969	105.2	0.227	0.687	16.94
Diazinon	0.9998	101.2	0.039	0.118	4.97
Endosulfan I	0.9987	99.4	0.145	0.439	5.87

**Table 2:** Khat chewing behaviors among Khat chewers in Amhara region, North West Ethiopia 2019 (*N* = 478).

Variables		Frequency	Percentage
Sex	Male	450	94.1
	Female	28	5.9

Age	18–24	88	18.4
	25–30	223	46.7
	31–35	82	17.2
	36–40	52	10.9
	41–45	18	3.8
	>45	15	3.1

Duration of Khat chewing/year	1–5	170	35.6
	6–10	182	38.1
	11–15	80	16.7
	16–20	34	7.1
	>20	12	2.5

Average duration of Khat chewing in min/day	15–90	89	18.6
	91–180	98	20.5
	>180	291	60.9

Amount of gram in range	25–75	143	29.9
	100–200	270	56.5
	225–500	65	13.6

Number of days/week	Daily	275	57.5
	4–6 days	68	14.2
	2-3 days	109	22.8
	Once	26	5.4

**Table 3:** The average concentration and European Union Maximum Residue Level (EU-MRL) of common pesticides used for Khat cultivation in Amhara region, North West Ethiopia, 2019.

Pesticide residue type	Average conc. (*μ*g/kg)	No of samples with pesticide residue (%)	EU-MRL (*μ*g/kg) [25]
Chlorpyrifos	75.4 ± 20.3	28 (93.3)	10
Dimethoate	130 ± 48.9	24 (80)	50
Profenofos	242.5 ± 119.8	6 (20)	50
Diazinon	81.5 ± 21.0	2 (6.6)	50

**Table 4:** Estimated daily intake, hazard quotient, and acute/short-term health risk index of pesticide residues by Amhara people from Khat chewing. The ADI and ARfD were obtained from the IUPAC pesticides properties database [26].

Pesticide	Av. conc	Max. conc	EDI	ADI	HQ	ESTI	aRfD	aHI (%)
Chlorpyrifos	75.4	173.23	0.24	5	0.05	0.56	1	55.61
Dimethoate	130	463.46	0.42	1	0.42	1.49	10	14.88
Profenofos	242.5	449.7	0.78	30	0.03	1.44	1000	0.14
Diazinon	81.5	102.5	0.26	0.2	1.31	0.33	25	1.32

EDI: estimated daily intake, HQ: hazard quotient, ADI: acute daily intake, aHI: acute health risk index, ESTI: estimated short-term intake, and aRfD: acute reference dose.

## Data Availability

The data used to support the findings of this study are included within the article.
